# Correlation between the timing of autonomous selfing and floral traits: a comparative study from three selfing *Gentianopsis* species (Gentianaceae)

**DOI:** 10.1038/s41598-018-21930-9

**Published:** 2018-02-26

**Authors:** Ji-Qin Yang, Yong-Li Fan, Xian-Feng Jiang, Qing-Jun Li, Xing-Fu Zhu

**Affiliations:** 1 Key Laboratory of Tropical Forest Ecology, Xishuangbanna Tropical Botanical Garden, Chinese Academy of Sciences, Mengla County, Yunnan 666303 China; 2 Gansu Liancheng National Nature Reserve, Yongdeng County, Gansu 730333 China; 3 Laboratory of Ecology and Evolution Biology, State Key Laboratory in Conservation and Utilization of Bioresources in Yunnan, Yunnan University, Kunming, Yunnan 650091 China

## Abstract

About 20% of angiosperms employ self-fertilization as their main mating strategy. In this study, we aimed to examine how the selfing timing correlated with floral traits in three *Gentianopsis* species in which autonomous selfing is achieved through filament elongation. Although the three *Gentianopsis* species exhibit no significant variation in their capacity for autonomous selfing, flowers of *G*. *grandis* last longer, are larger and have a higher corolla biomass, P/O ratios and male biomass allocation than those of *G*. *paludosa*, and especially those of *G*. *contorta*. Autonomous selfing occurs in the early floral life of *G*. *paludosa* and *G*. *contorta* and in the later floral life of *G*. *grandis*. Seed production mainly results from autonomous selfing in *G*. *paludosa* and *G*. *contorta*; however, *G*. *grandis* could be more described as having a mixed mating system. We suggest that autonomous selfing in later floral life increases the chance of cross-pollination prior to this, while autonomous selfing in early floral life offers a selective advantage to plants by reducing the resource investment in traits that may increase pollinator attraction and visitation.

## Introduction

Understanding how plants ensure reproductive success is important in plant evolutionary biology^1^. Plants have evolved various strategies to ensure successful reproduction in diverse environments^2^. Around 20% of flowering species employ self-fertilization as their main mating strategy^3^. Selfing plants have a 50% gene transmission advantage compared to outcrossing plants^4–6^, but their offspring may have low fitness because of inbreeding depression and the costs of gamete and seed discounting^4,7–9^. Selection for reproductive assurance, where selfing guarantees seed production when a lack of pollinators or inefficient pollen transfer limit reproductive success, is the most widely accepted hypothesis explaining the evolution of selfing^10,11^.

In autonomous selfing, the selfing time is a key trait which is generally under the nature selection^12,13^. There are three categories of autonomous selfing: prior, competing and delayed self-pollination. These occur, respectively, before, during and after opportunities for outcrossing^13,14^. If pollinators are sometimes unreliable, delayed selfing tends to be the favored type^15–17^. Delayed selfing does not interfere with outcrossing and functions as a “better than nothing” alternative even when it results in high inbreeding depression^18^. Under such conditions, cross-pollination can occur in early floral life, whilst the delayed selfing guarantees successful pollination at the end of the flower’s life if outcrossing fails^13,19,20^. If there are no pollinators, competing even prior selfing may offer a selective advantage over delayed selfing, by reducing the resources invested in traits associated with attracting pollinators and optimizing visits, for example floral longevity, floral size and nectar production^21–23^. There is variation in the timing of autonomous selfing even in closely related species^12,24^ or different populations of a single species^25–27^. This provides opportunities to evaluate the evolutionary significance of different types and timings of autonomous selfing.

*Gentianopsis* is a genus of flowering plants in the gentian family, commonly known as the fringed gentians; they have blue to purple flowers. Delayed selfing has been reported in two species of *Gentianopsis* (*G*. *barbata* and *G*. *paludosa*) on the Qinghai-Tibetan Plateau; in both cases this is achieved by elongation of the filaments as the flower ages^28,29^. *Gentianopsis ciliata*, which is found in central Europe, is self-compatible but hardly ever sets seed under natural conditions due to pollen limitation, indicating that this species has not evolved an autonomous selfing mechanism^30,31^. More studies are needed to understand the evolution of mating systems in this genus. Here, we compared variations in the timing of autonomous selfing in order to examine how various floral traits are adapted in three species of *Gentianopsis* (*G*. *contorta*, *G*. *paludosa* and *G*. *grandis*,) in NW Yunnan, China. We address the following questions: (1) Are all the three *Gentianopsis* species capable of autonomous selfing? If so, then (2) how are floral traits correlated with the timing of autonomous selfing?

## Materials and Methods

### Study species and populations

**Figure 1:**
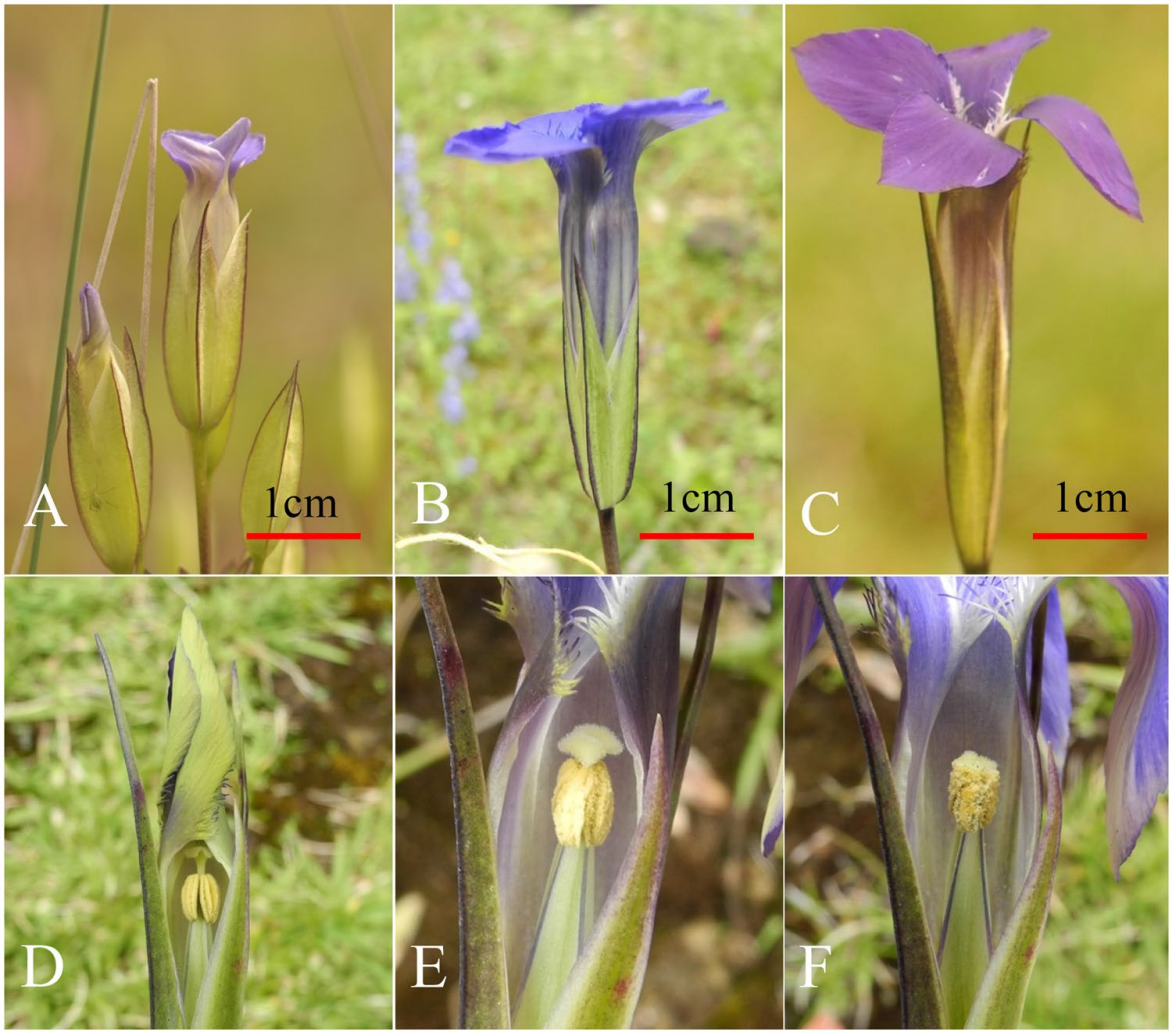
Flowers of *Gentianopsis contorta* (**A**), *G*. *paludosa* (**B**), *G*. *grandis* (**C**) and flower development of *G.* *grandis* (**D**: flower at bud stage, **E**: flower on the second day, **F**: flower on the fourth day).

The genus *Gentianopsis* contains 24 species and is widely distributed across the North Temperate Zone. Five species are native to China and occur at altitudes from 700 m to 4900 m^32^. Three species of *Gentianopsis* found in Northwest Yunnan, China – *G*. *contorta*, *G*. *grandis*, and *G*. *paludosa* – were selected for our study. The timing of flowering differs between the three, with *G*. *contorta* and *G*. *grandis* flowering from the beginning to the end of September, and *G*. *paludosa* flowering from the end of August to the middle of September. All the three species are characterized by a long blue to purple corolla tube, four horizontally spreading lobes and four projecting nectaries, which are positioned at the base of the corolla lobe (Fig. 1).

This study was conducted between early August and early October in 2012 and 2013. The population of *G*. *contorta* was at the Shangri-La Alpine Botanical Garden, Yunnan, China (27.9050°N, 99.6364°E; altitude, 3300 m a.s.l.). Pollination experiments involving *G*. *paludosa* were carried out at Potatso National Park, Shangri-La Yunnan, China (27.8588°N, 99.9952°E; altitude, 3600 m a.s.l.). The *Gentianopsis grandis* studied was in Xiaozhongdian, Shangri-La, China (27.5672°N, 99.8060°E; altitude, 3250 m a.s.l.). All three sites are species-rich meadows. Floral traits and the autonomous self-pollination mechanism of the three *Gentianopsis* species were studied during their peak flowering seasons.

### Floral traits

Thirty-six to forty-one plants of each species were randomly selected for a survey of the number of flowers per plant. We recorded eight floral traits: flower longevity, corolla tube length, corolla diameter, pollen production per flower, number of ovules per flower, pollen/ovule ratio, male investment/female investment and corolla biomass. In general, we selected apical flowers or floral buds from different individuals of each species for the measurements. To record flower longevity, one unopened bud of 34 to 41 individuals of each species was labeled. We observed these flowers twice a day, in the morning (ca. 08:00) and the evening (at 20:00). The length of the corolla tube and corolla diameter were measured on about twenty flowers per species using a hand-held digital caliper. We also quantified pollen grain and ovule numbers for the three species by selecting thirty flower buds, dissecting the anthers and ovaries and preserving them in 70% alcohol. Pollen production per flower was estimated following the method of Dafni^33^. The number of ovules in each ovary was counted under a dissecting microscope. For each flower, the pollen/ovule (P/O) ratio was calculated as the number of pollen grains divided by the number of ovules. We also dissected about 30 flower buds of each species, separating the pistil, stamens and corolla before determining their dry weights. All the floral parts were oven-dried for a minimum of 48 h at 80 °C and were weighed to the nearest 0.0001 g. We calculated the male investment/female investment (androecium biomass/gynoecium biomass), to represent the male sex allocation^34,35^. Floral traits were compared between species using one-way ANOVA with species as the fixed factor.

### Flower development

To investigate any change in herkogamy during flower opening, 20 flower buds from different plants of each species were selected and labeled. At the flower bud stage, we removed one lobe of the corolla and each day we measured the heights of the stamens and pistil using a hand-held digital caliper; these measurements continued each day until the flowers withered. Duan *et al*.^29^ demonstrated that the removal of a flower lobe in this way does not affect the process of flower development. The heights of the stamens and the pistil were measured from the bottom of the ovary, and the distance from the stigma to the anthers was calculated by subtracting the height of the stamens from the height of the pistil.

### The capacity for autonomous self-fertilization

To investigate each species’ capacity for autonomous selfing, we selected 31 to 33 individuals of each and enclosed their inflorescences within fine nylon nets. We performed three pollination treatments for each individual at peak flowering: (1) a flower that was not emasculated, which produced seed as a result of autonomous selfing; (2) a flower that was emasculated prior to flower-opening, but which was pollinated using pollen from other flowers on the same parent plant; and (3) a flower that was emasculated prior to flower-opening. The final treatment (3) was employed to test whether the three *Gentianopsis* species are able to produce any seeds through apomixis. Fruits were collected once seeds were ripe and seed set was evaluated as the number of seeds divided by the total number of ovules for each flower. Following Lloyd and Schoen^14^, the index of autonomous selfing [i.e. autofertility (AF)] was calculated as follows: [(mean autonomous seed set of unmanipulated flowers)/(mean seed set of supplemental pollinated flowers)]. Two-way ANOVA was performed to examine the effects of treatment and species on seed set.

### The timing of autonomous self-fertilization

The timing of autonomous selfing was investigated by emasculating flowers at different points in the flowering period. According to the floral longevity of each species, the following treatments were applied to about 30 flowers per treatment: anther removal (1) at the day before the first day that the flower was open; (2) at the first day that the flower was open; (3) at the second day that the flower was open, until the end of the flowering day, and (4) a control treatment in which the anthers were left intact. All the fruits were collected before dehiscence and taken back to the laboratory for seed set to be evaluated.

### Reproductive assurance

To assess the degree of reproductive assurance, we selected 27 to 41 individuals of each species. A flower from each individual was emasculated before anther dehiscence, and another flower was left intact and unmanipulated as a control. Both treatments were left for open pollination. For each fruit, seed set was determined in the laboratory as the number of seeds/the total number of ovules. Reproductive assurance was estimated as: 1 − (seed set of emasculated flower/seed set of intact flower)^4,11^. One-way ANOVA with species as a fixed factor was used to compare the difference in the magnitude of reproductive assurance.

## Results

### Floral traits

There was no statistically significant difference in the total number of flowers that the three *Gentianopsis* species produced per plant (F_2,114_ = 1.29, *P* = 0.28), although *G*. *paludosa* had the largest number per plant, whilst *G*. *contorta* had the fewest (see Table 1). The *Gentianopsis* flowers opened from 0900 h and closed at about 1700h every day due to the drop in temperature during the night; on rainy days the flowers closed temporarily and flower life was increased. Flowers lasted an average of 4.05 ± 0.11, 4.58 ± 0.33, 6.25 ± 0.28 days, respectively, for *G*. *contorta*, *G*. *paludosa* and *G*. *grandis*, and the floral longevity in the three species was significantly different (F_2,107_ = 4.42, *P* < 0.05). The *Gentianopsis* species differed significantly in flower size (corolla tube length: F_2,59_ = 394.30, *P* < 0.001; corolla diameter: F_2,59_ = 128.90, *P* < 0.001; corolla biomass: F_2,92_ = 18.73, *P* < 0.001), with *G*. *grandis* producing the largest and heaviest flowers, *G*. *paludosa* being intermediate, and *G*. *contorta* producing the smallest and lightest flowers. The three species also differed significantly in the number of ovules (F_2,46_ = 65.22, *p* < 0.001) and pollen grains (F_2,46_ = 37.88, *P* < 0.001) produced per flower. *Gentianopsis contorta* produced the largest number of ovules and an intermediate number of pollen grains, while *G*. *grandis* produced the largest number of pollen grains and an intermediate number of ovules. The P/O ratio varied significantly between the three species (F_2,46_ = 40.59, *P* < 0.001), with *G*. *grandis* having the largest ratio, *G*. *paludosa* being intermediate and *G*. *contorta* having the smallest. The male sex allocation also differed significantly between the three species (stamens biomass/pistil biomass: F_2,92_ = 31.74, *P* < 0.001), with *G*. *grandis* allocating more resources to male function (stamens), and *G*. *contorta* allocating more resources to female function (pistil).

**Table 1:** Floral traits of the three *Gentianopsis* species, values with different letters in the same column indicate significant differences at the 0.05 level. Numbers in parentheses are sample sizes.

Floral characteristics	*G*. *contorta*	*G*. *paludosa*	*G*. *grandis*
No. of flowers/Plant	5.5 ± 0.6^a^ (40)	6.9 ± 0.7^a^ (36)	6.4 ± 0.6^a^ (41)
Floral longevity (d)	4.58 ± 0.33^a^ (34)	4.05 ± 0.11^a^ (35)	6.25 ± 0.28^b^ (41)
Corolla tube length (mm)	23.40 ± 0.68^a^ (30)	35.38 ± 0.50^b^ (30)	41.89 ± 0.83^c^ (30)
Corolla diameter (mm)	14.24 ± 0.59^a^ (30)	21.31 ± 0.40^b^ (30)	30.14 ± 0.94^c^ (30)
Pollen grain number	22796 ± 1638.95^a^ (19)	17685.71 ± 1379.84^b^ (16)	34672.50 ± 1379.84^c^ (16)
Ovule number	3772.37 ± 290.88^a^ (19)	735.20 ± 46.14^b^ (14)	1411.83 ± 70.92^c^ (16)
P/O ratio	6.48 ± 0.61^a^ (19)	25.28 ± 2.03^b^ (14)	26.00 ± 2.27^b^ (16)
Stamen biomass/Pistil biomass	0.42 ± 0.11^b^ (31)	0.54 ± 0.10^a^ (30)	0.60 ± 0.17^a^ (34)
Corolla biomass (g)	0.0287 ± 0.0062^a^ (31)	0.0323 ± 0.0039^a^ (30)	0.1060 ± 0.0153^b^ (34)

### Flower development

**Figure 2:**
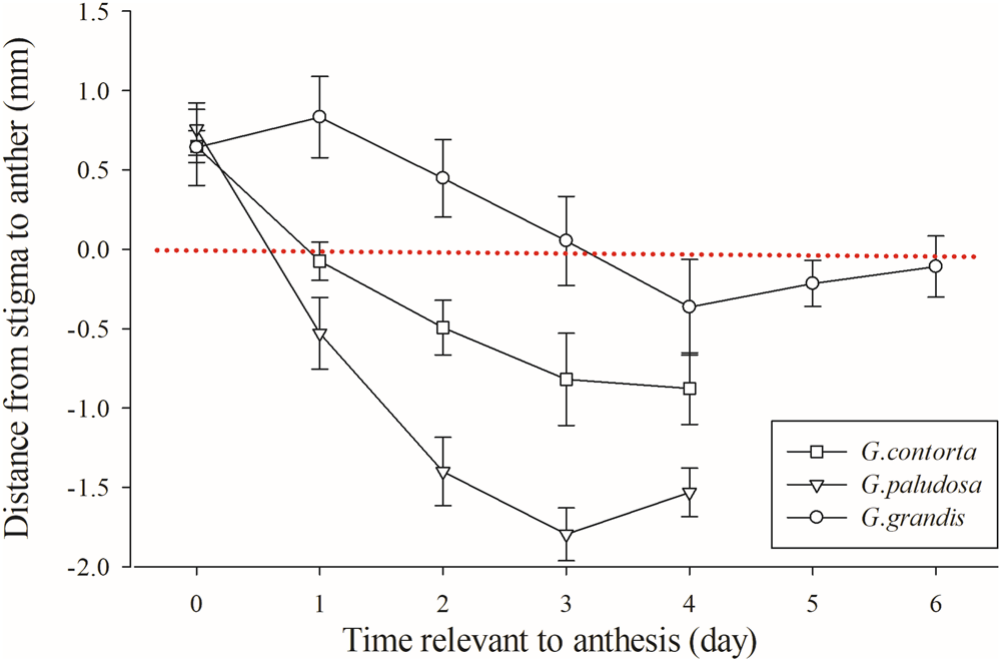
Change of distance from stigma to anther in relation to flower opening in three *Gentianopsis* species.

We found the degree of herkogamy (the distance between the stamens and pistil) changed during the flower’s development in the three species, mainly resulting from the elongation of filaments (Figs 1 and 2). At the floral bud stage, the stamens were shorter than the pistil, and then the stamens elongated as the flower developed. The height of the anthers gradually equaled or exceeded the height of the stigmas, resulting in stigma-anther contact. We observed flowers with stigma-anther contact on the first day of flower opening for *G*. *contorta* and *G*. *paludosa*, but it was about three days after anthesis commenced that the height of the anthers equaled the height of the stigmas in *G*. *grandis*.

### The capacity for autonomous self-fertilization

None of the bagged flowers that had been emasculated before anther dehiscence produced any seeds, indicating that there was no apomixis in the three *Gentianopsis* species. There was no difference in seed set between autonomous selfed flowers and hand self-pollinated flowers (F_2,190_ = 0.06, *p* = 0.80), nor were the values affected by the treatment and species interaction (F_2,190_ = 0.37, *p* = 0.70); however, there was a significant difference between species (F_2,190_ = 23.99, *p* < 0.001). The three *Gentianopsis* showed no significant variation in their capacity for autonomous selfing (F_2,95_ = 1.17, *P* = 0.31).

### Timing of autonomous selfing

**Figure 3:**
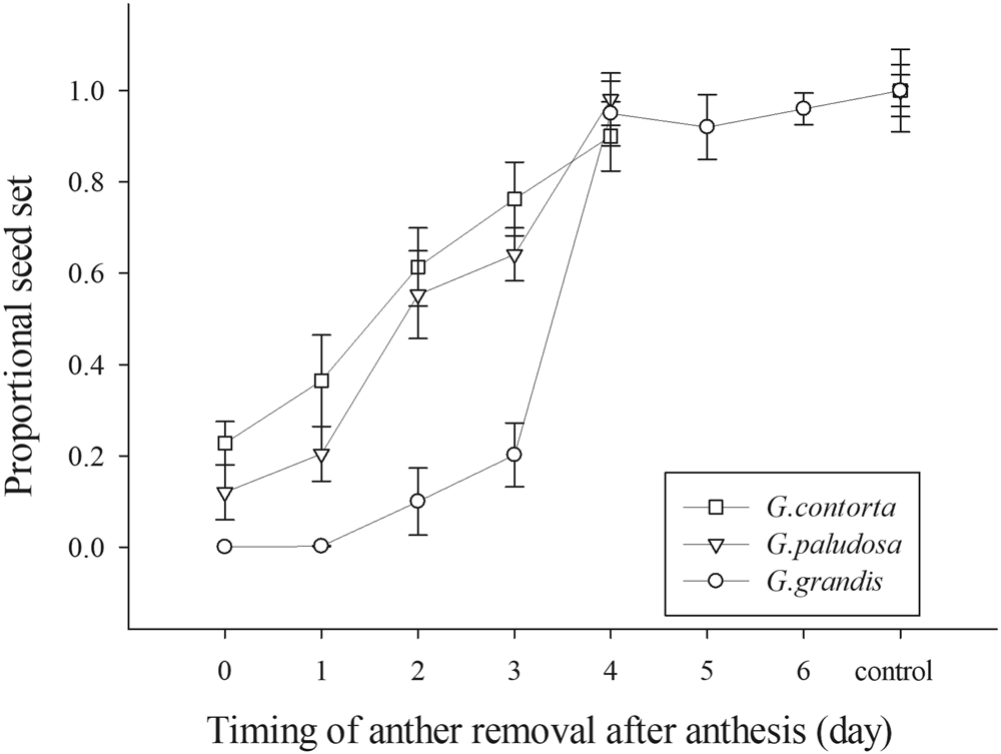
The relationship between the timing of anther removal and seed production by autonomous selfing in *Gentianopsis contorta*, *G*. *paludosa* and *G*. *grandis*. Flowers were bagged for the entire flowering period.

In each of the three study species, the amount of seed set resulting from autonomous selfing increased significantly with flower age, but the rate of increase differed significantly between the species (Table 2). *Gentianopsis paludosa* and *G*. *contorta* achieved half of their total autonomous seed set by the second day of flowering, whereas the rate of increase in autonomous selfing peaked much later in *G*. *grandis* and on the fourth day all flowers suddenly set seed as control (Fig. 3).

**Table 2:** The effects of species (*G*. *contorta*, *G*. *paludosa* and *G*. *grandis*) and timing of emasculation on autonomous seed production. Asterisks denote significant differences (***P < 0.001).

Source	*d*. *f*.	MS	F
Species	2	1.65	46.66^***^
Timing	6	4.25	117.34^***^
Timing × species	8	0.51	14.15^***^
Error	454	0.36

### Reproductive assurance

**Figure 4:**
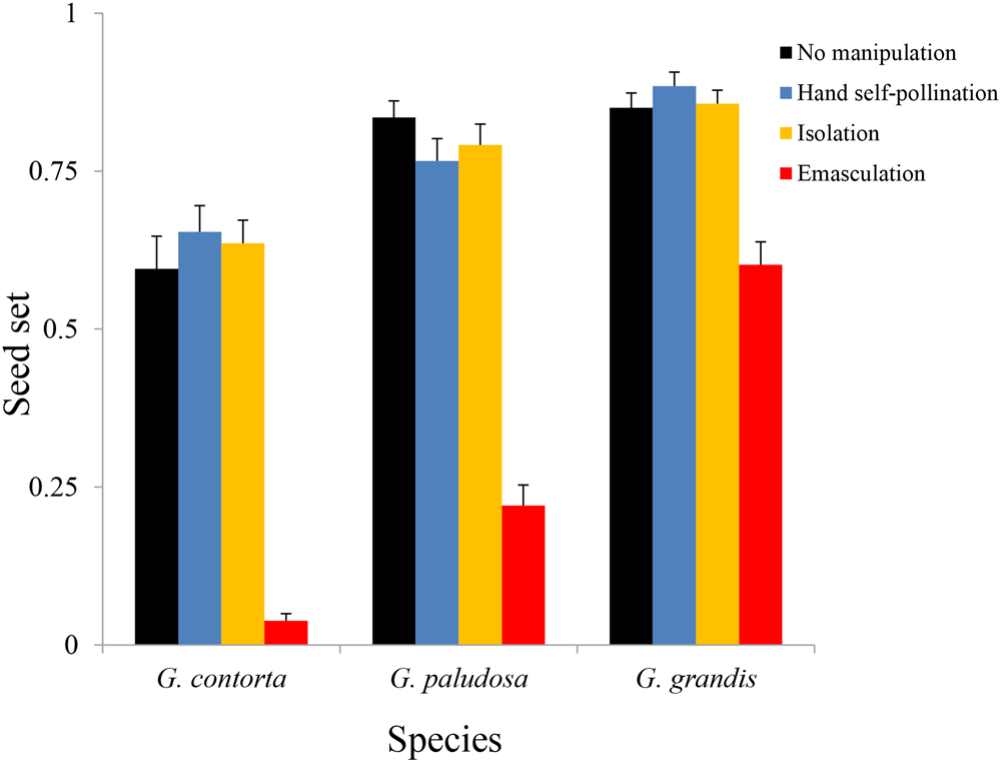
Seed set per flower after no manipulation, hand self-pollination, isolation, and emasculation in the three *Gentianopsis* species.

In all three species, the seed set of the control was significantly higher than when flowers were emasculated (*G*. *contorta*: *t* = 10.47, *d*. *f*. = 26, *P* < 0.001; *G*. *paludosa*: *t* = 10.47, *d*. *f*. = 40, *P < *0.001; *G*. *grandis*: *t* = 16.08, *d*. *f*. = 37, *P* < 0.001), indicating that autonomous self-pollination delivers reproductive assurance in all three species (Fig. 4). The three species showed significant variation (F_2,103_ = 84.75, *P* < 0.001) in their capacity for reproductive assurance (RA = 0.91 ± 0.03, 0.73 ± 0.04 and 0.28 ± 0.04 for *G*. *contorta*, *G*. *paludosa*, and *G*. *grandis* respectively).

## Discussion

The changes over time in the relative positions of stigmas and anthers within flowers of self-compatible plants are important in controlling the timing of autonomous selfing^11,36^. As with previous studies^28,29^, our research suggests that the three *Gentianopsis* species (*G*. *contorta*, *G*. *paludosa* and *G*. *grandis*) have the capacity to self autonomously mainly through filament elongation, but the timing of this differs between the species. At the floral bud stage, the stamens are shorter than the pistil in all three species. Stigma and anthers within flowers came into contact with each other on the first day of flower opening in *G*. *contorta* and *G*. *paludosa* mainly because of filament elongation, while the height of the anthers equaled the height of stigmas about three days after anthesis commenced in *G*. *grandis* (Fig. 2). The data from our emasculation experiments indicated that flowers are autonomously fertilized in the early floral lifespan in *G*. *contorta* and *G*. *paludosa*, suggesting that the timing of autonomous selfing in the two species can certainly be considered to indicate competing selfing, whilst in *G*. *grandis* the autonomous selfing occurs in the late floral stage and tends to represent delayed selfing (Fig. 3).

In our study, flowers of *G*. *grandis* lasted longer and were significantly larger with higher corolla biomass, P/O ratios and male allocation (stamens biomass/pistil biomass) than those of *G*. *paludosa*, and especially those of *G*. *contorta* (see Table 1). These results support the idea that the later selfing species retain the floral traits and pay the costs associated with attracting polliantors^19,37^. Indeed, we frequently observed bumblebee pollinators effectively visiting flowers of *G*. *grandis*, whilst only two and one bumblebee visits, respectively, were observed in *G*. *paludosa* and *G*. *contorta* during the experimental period. The emasculation experiments showed 71%, 27% and 9% of the ovules were fertilized by outcrossing pollen grains for *G*. *grandis*, *G*. *paludosa*, and *G*. *contorta*, respectively (Fig. 4). Although the three *Gentianopsis* species exhibited no significant variation in their capacity for autonomous selfing, seed production mainly resulted from autonomous selfing in the two competing selfing species, especially *G*. *contorta*. *Gentianopsis grandis* could be characterized as having more of a mixed mating system, producing offspring by autonomous selfing when pollinator-mediated pollination fails.

Duan *et al*.^29^ found that anthers made contact with the central stigma and self-pollination occurred at the end of the floral lifespan in *G*. *paludosa* on the Qinghai-Tibetan Plateau, which is typical of delayed selfing. However, we found that flowers of *G*. *paludosa* were autonomously fertilized in the early floral lifespan and that stigma-anther contact occurred once the flower had opened in plants from Northwest Yunnan. These findings suggest that even within the same species the timing of autonomous selfing may exhibit substantial variation, and such differences may in turn contribute variable amounts of autonomously selfed seeds to total seed set. Further studies are needed to explore how evolution history and ecological factors shape the autonomous selfing traits within *Gentianopsis* species.

In all the three *Gentianopsis* species, anther-stigma contact did not indicate that autonomous selfing would result in a large amount of seed production immediately. The anthers were already in contact with the stigma on the first flowering day for *G*. *contorta* and *G*. *paludosa*, while the amount of seed set resulting from autonomous selfing increased linearly with flower age. We observed stigma-anther contact on the third day of flower opening in *G*. *grandis*, but autonomous selfing mainly occurred on the fourth day (Figs 2 and 3). The fact that the timing of autonomous selfing was later than the timing of anther-stigma contact in the three species may result from the gradual exposure of pollen grains on anthers and low P/O ratios in the three *Gentianopsis* species. In general, pollen grains on anthers gradually become susceptible to removal, thereby preserving pollen grains on the anthers when there are few pollen vectors and/or restricting pollen removal by individual pollinators, so that many pollinators can be involved in pollen dispersal^38,39^. Furthermore, the P/O ratios of the three species are quite low (6.48 ± 0.61, 25.28 ± 2.03, 26.00 ± 2.27 respectively for *G*. *contorta*, *G*. *paludosa* and *G*. *grandis*). Although anthers dehisce once flowers open in the three species, only some of the pollen grains could touch and germinate on stigmas when anther-stigma contact occurs; thus, only a small proportion of ovules would be fertilized. All three *Gentianopsis* species still have some chance of outcrossing since the flowers set at least some seeds after emasculation (Fig. 4). It would be worth examining the mating system of the three *Gentianopsis* species using molecular makers to determine whether the interval between the anther-stigma contact and the actual occurrences of autonomous selfing allows the opportunity for stigmas to receive outcrossing pollen grains.

## Conclusion

Our results clearly indicate that autonomous selfing in later floral life preserves the chance of cross-pollination in early floral life and that autonomous selfing in early floral life offers a selective advantage to plants by reducing their investment in traits that may increase pollinator attraction and visitation. Flowers are autonomously fertilized in the early floral lifespan in *G*. *contorta* and *G*. *paludosa*, but in *G*. *grandis* autonomous selfing occurs later. The three *Gentianopsis* species show no significant variation in their capacity for autonomous selfing, although flowers of *G*. *grandis* last longer and are larger with higher corolla biomass, P/O ratios and male biomass allocation than those of *G*. *paludosa*, and especially those of *G*. *contorta*. We also found that the timing of autonomous selfing was later than the timing of anther-stigma contact, probably resulting from the low P/O ratios and the gradual exposure of pollen grains on anthers in the three *Gentianopsis* species, although plants with obligate autogamous mating systems usually have low P/O ratios^40,41^.
